# Progress in Medicine

**Published:** 1899-02-11

**Authors:** 


### Progress in Medicine.

### NEUROLOGY.

(*Continued from page* 315.)

The differential diagnosis between progressive dementia and syphilitic brain disease resembling it calls for careful discussion. The problem is all the more difficult because syphilis is so often a factor in the production of progressive dementia. Symptoms which point to the existence of a general cerebro-spinal syphilis (such as pupillary immobility, ocular palsies, preceding and transitory apoplexies) are the most valuable signs in diagnosis.

It is worthy of remark that whilst paretic dementia does occur, though rarely, in younger individuals, yet delusions of grandeur associated with the usual physical symptoms, if occurring in young individuals, need not portend the development of this dreaded disease.

Patrick[3] also has carefully discussed the points of distinction between cerebral syphilis and general paralysis of the insane. By cerebral syphilis is meant lesions of this organ that are universally recognised histologically as belonging to the direct pathological changes induced by metic infection, viz., syphilitic arteritis, and meningitis, and gumma, and conditions immediately secondary to these. The mere fact of previous specific infection is of no real value, and likewise absence of syphilitic history is to be given no weight. But if there is a history of infection, the lapse of time between the primary sore and the appearance of brain symptoms is of some importance, for brain syphilis in the vast majority of cases does not come on at a remote period after infection; more than half of the cases appear within three years. On the other hand, the length of time a syphilitic has to wait for general paralysis, supposing this disease to supervene, is distinctly more extended; in over 72 per cent. of cases, ten years or more had elapsed since the chancre, and only 3 per cent. occurred within five years.

As regards the clinical phenomena, it is generally found that a case of brain syphilis which resembles paralytic dementia presents a good clinical picture of the latter disease, with something added, and this something is more frequently of a somatic than psychic character.

The very earliest symptoms of the two diseases are, as a rule, widely different. The first signs of dementia paralytica are almost exclusively psychic, dulling of the ethical sense, slowing of complex mental processes, impaired judgment, lack of attention, impaired memory. Similarly the motor disturbance of paralytic dementia involves first the most highly evolved functions, those most intimately connected with psychic activity, speech, singing, facial expression, the nicer co-ordinate movements. In syphilis of the brain, on the other hand, the inception is nearly always marked by somatic disturbance—weakness, from that of an ocular muscle to a hemiplegia, paræsthesia, vertigo, and a fit of any kind.

General paralysis is insidious in its commencement and progressive in its course. Temporary arrest is not very rare, but intermissions, returns to normal, are almost unknown. On the other hand, brain syphilis may have a gradual beginning, but usually there is something of the sudden and unexpected in its onset, and the same characters are true of its further course.

In cerebral syphilis, whatever the particular lesion, headache is tolerably certain to be present. It is intense, persistent, generally nocturnal, often localised, and accompanied by tenderness to percussion. On the other hand, intense cephalagia does not occur in paralytic dementia, even when there is co-existent arterio-sclerosis. A feeling of discomfort or pressure —a cephalic distress—is frequent, but it is quite a different thing from the headache of cerebral syphilis. Distinct visceral or peripheral neuralgias very strongly point to syphilis as opposed to dementia paralytica. A general paralytic may, of course, suffer from various aches and pains, but these are not of the burning, stabbing, and boring character of those which are syphilitic in origin. An exception to this rule is that a paralytic may also have tabes with its characteristic tightening or boring pains. Insomnia may occur both in cerebral syphilis and paralytic dementia. Amongst the greatest aids in a differential diagnosis are those symptoms due to involvement of the cranial nerves. In general paresis any involvement of these nerves below the third, except possibly the sixth, is rare, whilst in syphilis, although the third is the one most frequently affected, none is exempt, and multiple nerve palsies are frequent. An Argyl Robertson pupil is strongly suggestive of general paresis being present in from 60 to 80 per cent. of cases; whilst it is very rare in cerebral syphilis. With remarkably few exceptions, simple optic atrophy means general paresis, and optic neuritis means syphilis. Transient or recurring amblyopia, hemiopia, or other segmental defect in the visual field, are strongly in favour of syphilis. Facial paralysis, anæsthesia in the distribution of the fifth nerve, or atrophy of the tongue are generally syphilitic. Similarly, a crossed paralysis of a cranial nerve and the opposite side of the body is nearly always syphilitic in origin.

In regard to the psychic symptoms of cerebral syphilis and general paralysis, it is first to be remarked that neither disease ever produces a psychic symptom not to be found in the other, but there is a difference in the mode of onset. General paralysis shows itself insidiously, attacking first the higher faculties, and only gradually invading the lower. On the other hand, in cerebral syphilis there is a more general hebetude, and the disease in its onward march lacks uniformity of movement, becoming worse by fits and starts; and it may make its appearance even suddenly, as by a gradually deepening stupor. In some cases of general paralysis there may be mental depression which may closely simulate melancholia, and in other cases there may be marked mental eagerness and restlessness, which is never found in syphilis. A long course of gradually increasing dementia, even if marked by maniacal outbreaks or temporary aggravations following convulsive seizures, is characteristic of general paralysis, whilst in syphilis, even if there be dementia, there is added to it a degree of irritability, excitation, apathy, or stupor incompatible with paretic dementia, and, moreover, this dementia may suddenly improve and be replaced by, *e.g.*, mania or delirium. Not too much weight should be given to the absence of delusions of grandeur in the diagnosis of paralytic dementia, as they are often absent. Such delusions, if present, are not pathognomonic of general paralysis, for they may occur in cerebral syphilis. In tumours of the prefrontal lobe, too, whatever their origin, there is often a tendency to illogical cheerfulness or hilarity, with an inclination to be facetious. On the other hand, fatuous delusions of grandeur, with absence of all signs of gross brain disease, is strong evidence in favour of general paralysis. A cerebral syphilitic is apt to comprehend his condition better than a paretic dement, though it is by no means true to say that the latter, at least in the early stages, is not conscious of any physical or mental disability. Both conditions are liable to maniacal outbreaks, but that of syphilis is apt to be simply an exhibition of excitement or brute violence, whilst in general paralysis delusions of grandeur and rapid play of ideas are almost sure to accompany the attack of mania. Intense hallucinations of the special senses are rather more characteristic of syphilis than of general paresis. The last resource in diagnosis is the effects of treatment, and sometimes valuable information may be obtained in this way, and if there is a doubt in the diagnosis the patient should be given it. This test is sometimes unreliable by reason of insufficient dosage, or of the presence of syphilitic lesions which cannot be cured by drugs, *e.g.*, softening due to arterial occlusion, and, again, in some cases of general paralysis improvement may result from anti-syphilitic treatment.

**Aphasia and Testamentary Capacity** were discussed by Gairdner[4] and by Elder[5] at the Edinburgh meeting. When a will is produced which is made in proper form and signed by the testator either in writing or by a mark witnessed by two persons, the law assumes it to be valid unless the contrary is shown. The grounds on which it is attacked are either testamentary incapacity or undue influence. Incapacity need not be absolute insanity, but has been defined to be "want of mind to understand" the will in question. An aphasic person, who was originally able to speak, and had learned to read and write, but who had lost the power of speech through accident or illness, would probably be considered capable of making a valid will unless it could be shown that his mental powers had been affected as well. The problem is very difficult when the patient, though apparently in possession of his mental faculties, lacks in a greater or less degree the means of expressing his ideas to others so as to make them intelligible. In such a case much would depend on the credibility of the witnesses, and on whether it duly provided for those to whom the testator would presumably be attached. On the other hand, if the accompanying symptoms were such as to lead to the inference that he was suffering from imbecility or mental debility as well as from loss of speech, there is very little doubt but that a will made by such a person would be held invalid. It results that no general rule can be laid down in regard to aphasiacs. The circumstances of their affliction vary greatly, and so does their testamentary capacity.

**Idioglossia.**—Guthrie[6] has reported an interesting case of this affection associated with pseudo-hypertrophic paralysis. The name idioglossia was given by Hale White and Golding Bird to a defective condition of speech, which gives the impression that the speaker uses a language of his own. The vocabulary of the case recorded consisted almost entirely of consonant sounds, b, p, t, d, t, k, and various vowel sounds. There was no defect in the vocal apparatus, but a defect of hearing short of being deaf. He had no ear for music. The mental condition was good, but there was a defective visual memory and want of attention. The condition is an exaggeration of defective modes of speech, which are extremely common in minor degrees without being regarded as in any way morbid. The difficulty these patients have in making themselves understood often gives the impression that they are imbeciles. But it is lack of ear, not of intellect, which prevents the idioglossic patient from speaking correctly. The treatment of this condition is entirely a matter of teaching, and the child should be taught to imitate by the eye and touch what he cannot do by the ear.

[3] Patrick, New York Med. Jour., Aug. 20, 27, Sept. 10, 17, 1898. [4] Gairdner, Brit. Med. Jour., Sept. 3, 1898. [5] Elder, Brit. Med. Jour., Sept. 3, 1898. [6] Guthrie, Clin. Jour., Aug. 10 and 17, 1898.

